# Representations of Codeine Misuse on Instagram: Content Analysis

**DOI:** 10.2196/publichealth.8144

**Published:** 2018-03-20

**Authors:** Roy Cherian, Marisa Westbrook, Danielle Ramo, Urmimala Sarkar

**Affiliations:** ^1^ Center for Vulnerable Populations Department of General Internal Medicine University of California, San Francisco San Francisco, CA United States; ^2^ Department of Health and Behavioral Sciences University of Colorado, Denver Denver, CO United States; ^3^ Weill Institute for Neurosciences Department of Psychiatry University of California, San Francisco San Francisco, CA United States

**Keywords:** prescription opioid misuse, social media, poly-substance use, Instagram

## Abstract

**Background:**

Prescription opioid misuse has doubled over the past 10 years and is now a public health epidemic. Analysis of social media data may provide additional insights into opioid misuse to supplement the traditional approaches of data collection (eg, self-report on surveys).

**Objective:**

The aim of this study was to characterize representations of codeine misuse through analysis of public posts on Instagram to understand text phrases related to misuse.

**Methods:**

We identified hashtags and searchable text phrases associated with codeine misuse by analyzing 1156 sequential Instagram posts over the course of 2 weeks from May 2016 to July 2016. Content analysis of posts associated with these hashtags identified the most common themes arising in images, as well as culture around misuse, including how misuse is happening and being perpetuated through social media.

**Results:**

A majority of images (50/100; 50.0%) depicted codeine in its commonly misused form, combined with soda (*lean*). Codeine misuse was commonly represented with the ingestion of alcohol, cannabis, and benzodiazepines. Some images highlighted the previously noted affinity between codeine misuse and hip-hop culture or mainstream popular culture images.

**Conclusions:**

The prevalence of codeine misuse images, glamorizing of ingestion with soda and alcohol, and their integration with mainstream, popular culture imagery holds the potential to normalize and increase codeine misuse and overdose. To reduce harm and prevent misuse, immediate public health efforts are needed to better understand the relationship between the potential normalization, ritualization, and commercialization of codeine misuse.

## Introduction

### Prescription Opioid Misuse

Prescription opioid misuse, defined by the National Institute on Drug Abuse as “the use of medication without prescription, in a way other than as prescribed, or for the experience or feelings elicited” [1], has doubled over the past 10 years [2]. The rapid rise of prescription opioid misuse has been identified as a key public health problem by the 2016 US Surgeon General [3] and is the subject of the bipartisan Comprehensive Addiction and Recovery Act signed by President Obama in 2016 [4]. To inform opioid misuse prevention and treatment efforts, public health leaders need a better understanding of the context, attitudes, and beliefs around these medications and their misuse.

### Substance Misuse and Social Media Research

Researchers and public health advocates can use social media data to provide insight into real-time attitudes, beliefs, and misuse of prescription opioids because diverse individuals share opinions, information, and images via social media [5-8]. Individuals’ posts on social media represent a lens through which researchers can examine risky behavior such as opioid misuse, while remaining unobserved themselves and thereby, mitigating the effects of observation bias [9-11]. Analysis of social media data may provide additional insights into opioid misuse to supplement the traditional approach of asking individuals to self-report behavior through surveys or interviews, similarly mitigating the effects of response bias [9].

Most studies characterizing prescription opioid misuse using social media have used text-based analysis of platforms such as Twitter [8,12]. For example, an analysis of Twitter conversations about nonmedical use of prescription opioids identified polysubstance use as a common theme, including combinations of classes of prescription drugs and illicit substances and the use of social media for trafficking prescription opioids [13,14]. Instagram, a primarily image-based tool, is the second most popular social media platform among teens, after Snapchat [15]. Given its prominence as a social media platform used to represent everyday life, Instagram-based research can help public health researchers discern attitudes and behaviors that may not be disclosed in more formal settings or through traditional research methodologies [16]. Instagram offers enhanced understanding of the functional context of and attitudes toward substance use through analysis of imagery, and it has been implicated as a more influential tool on substance use behavior than other forms of social media [17]. For example, one recent study categorized Instagram images of electronic cigarettes, highlighting the platform as a tool for supporting a vaping identity and for advertisers to reach a target audience of users [18]. Similarly, Cavazos-Regh et al describe how Instagram can offer insight into the patterns of marijuana use [19]. Furthermore, the sampling process is facilitated by the use of hashtags, words, or phrases preceded by the # symbol [20] that users incorporate in social media posts to contribute to, or create, an impromptu forum to discuss a specific topic or event by aggregating posts with the same hashtag.

In this paper, we sought to describe opioid misuse as depicted through images, videos, and captions publicly available on Instagram. We chose to focus on codeine because it has been hypothesized to be a gateway into opioid misuse and addiction [21]. Moreover, codeine misuse continues to increase despite rising costs for the drug and stricter regulations [22]. We chose to analyze images and videos on Instagram because it is the most popular social networking platform after Snapchat among US teens [15] who are the demographic group most at risk for initiating codeine misuse [21]. The purpose of this paper was to gain a better understanding of content related to codeine misuse as represented on social media to inform countermessaging and other public health efforts.

## Methods

### Study Design

**Figure 1:**
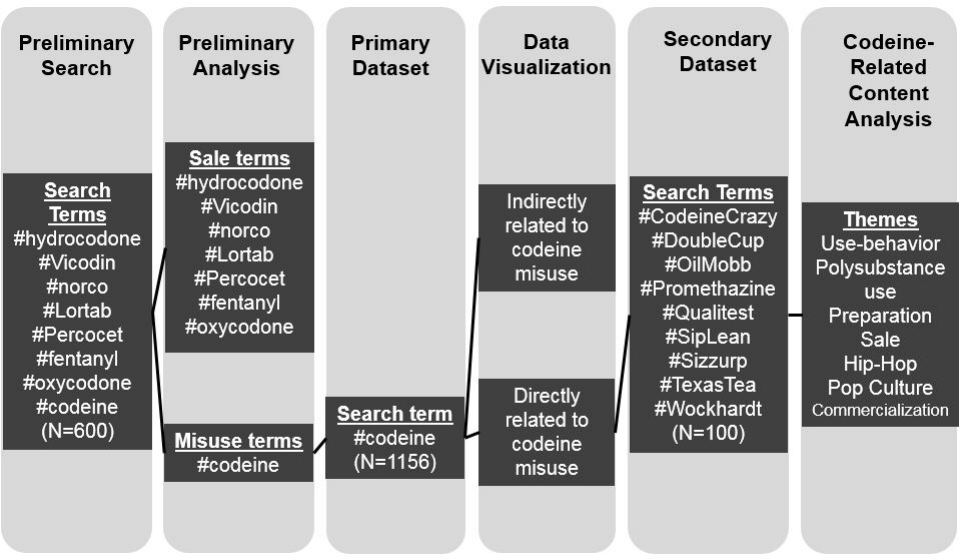
Study design.

We collected and content analyzed publically available, user-generated content about prescription opioid misuse posted to Instagram to understand the motivations and narratives related to uptake and misuse. As we used publically available data and did not collect or store identifying information, the *University of California, San Francisco* Institutional Review Board determined that this study did not require review. For preliminary analysis, we explored posts tagged with hashtags derived from generic and brand names of opioids (eg, *#vicodin*). Except for posts under #codeine, other generic and brand names were predominantly associated with sale. For primary analysis, we downloaded and analyzed all posts under *#codeine* for 1 week, listing all of the other hashtags associated with these posts. Among these, we downloaded the first 10 posts under the 10 most salient codeine-related hashtags for secondary analysis. Figure 1 visually represents our study design, which is described in detail below.

### Preliminary Search

A preliminary analysis of Instagram posts related to opioids revealed that virtually all used multiple hashtags. We manually collected screenshots and video captures of posts with opioid-related hashtags, starting first with generic and brand names (eg, *hydrocodone* and *vicodin*) for opioid medications and then iteratively expanding the search terms as we uncovered the hidden lexicon of opioid misusers. Researchers conducted analysis of videos and images by noting the most prominent features in images and using a narrative summary for the videos. To uncover the hidden lexicon, we noted all of the hashtags that were associated with the posts we collected under #codeine. From there, we used simple counts to determine which of these associated hashtags were most prominently associated with codeine misuse (Multimedia Appendix 1).

We downloaded screenshots and video captures of the 75 most recent Instagram posts for the eight most common opioid hashtags for this preliminary search (#hydrocodone, #vicodin, #norco, #lortab, #percocet, #fentanyl, #oxycodone, and #codeine) on May 27, 2016. We selected these eight hashtags as they are all well-known generic or brand names for opioids [6]. Preliminary analysis demonstrated that, respectively, 56% (42/75), 59% (44/75), 87% (65/75), 72% (54/75), 39% (29/75), 78% (59/75), and 84% (63/75) of posts related to the hashtags #hydrocodone, #vicodin, #norco, #lortab, #percocet, #fentanyl, and #oxycodone depicted photographs of loose pills or pill bottles and appeared to offer to sell opioids or were ambiguous. Posts with the hashtag #codeine, however, depicted varied images and text related to codeine misuse, including but not limited to codeine misuse being associated with cartoon characters, hip-hop artists, and larger lifestyle choices. Given the high level of variability of imagery and its hypothesized role as a gateway to opioid misuse and addiction [21], posts with the hashtag #codeine demonstrated a clear public health significance and were thus chosen for further content analysis.

Using the iterative sampling methods of grounded theory, we identified other opioid-related hashtags within *#codeine* posts to capture representations of codeine misuse that are not well known (eg, *#sizzurp* and *#oilmobb*) and identify slang terms for codeine, and thereby, understand how users represent misuse on Instagram [23]. The advantage of this approach is that it allows us to capture how users discuss codeine misuse on Instagram inductively and minimize the impact of our preconceived theories of misuse [23]. As the behaviors, as well as their associated meanings and representations tied to codeine misuse can change over time, it is important to have an agile, analytic approach capable of capturing not only preexisting patterns and narratives of misuse but new representations and subcultures as well.

### Primary Data Acquisition and Sampling

We manually downloaded posts with the hashtag *#codeine* each day of the week to account for daily variation, beginning on July 10, 2016, and ending on July 16, 2016. Weekends had more posts with the hashtag *#codeine*, resulting in a range of 150 to 200 posts downloaded each day of the week. We set thematic saturation at the point where we began to see duplicate posts. We downloaded a total of 1156 posts for our primary dataset.

### Primary Dataset: Data Visualization

**Figure 2:**
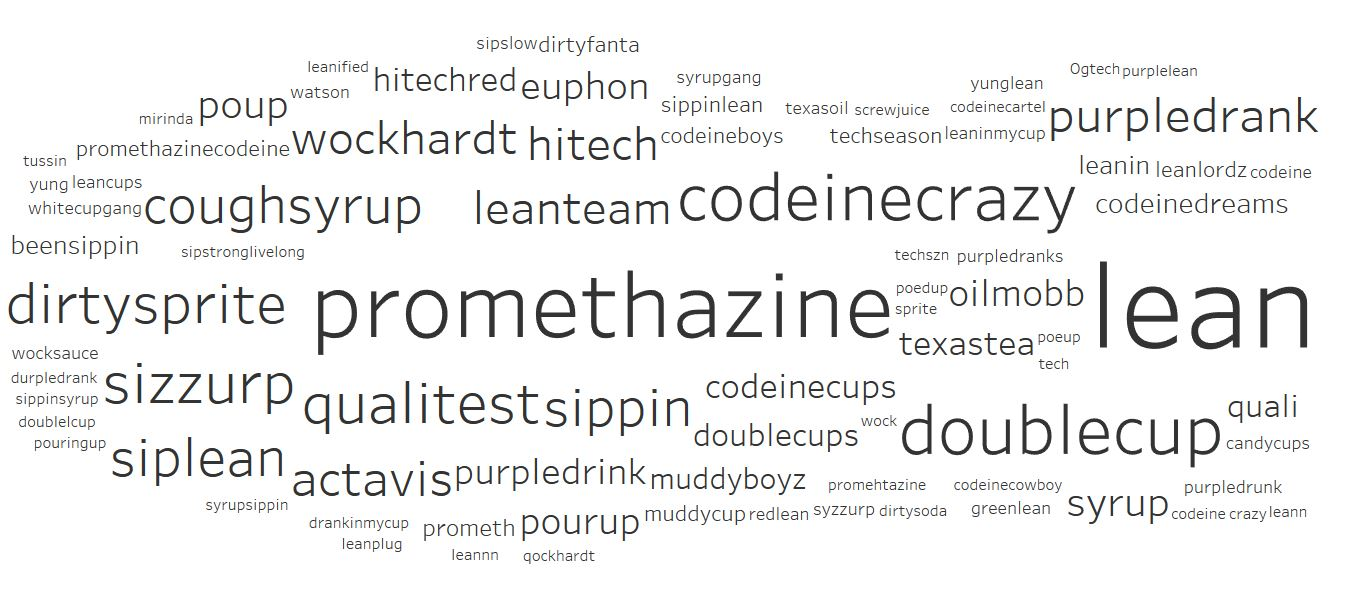
Most common opioid-related hashtags associated with #codeine.

The primary dataset comprised posts with the hashtag *#codeine*. For each post, we documented all listed hashtags and deductively designated them into lists of those that directly represented codeine misuse (eg, #CodeineCrazy) and those that did not (eg, #Trap) [24]. After sorting and counting terms directly and indirectly related to codeine misuse, we used Tableau (Tableau Software), a data visualization software, to demonstrate the relative salience of slang terms for codeine misuse, as well as other terms associated with codeine misuse (Figure 2). In these visualizations, the size of each term corresponds to its frequency in the dataset and helps represent the hidden lexicon and social milieu of codeine misuse, respectively. From the list of hashtags that directly relate to codeine-misuse, we derived search terms for our secondary dataset.

### Secondary Data Acquisition and Sampling

We used the 10 most common hashtags from the codeine-related list to identify a more focused sample of posts for our secondary dataset. A hashtag that was chosen had to have at least 1000 posts associated with it on Instagram and demonstrate codeine misuse in the top results shown by Instagram. Therefore, hashtags for secondary analysis not only had to be common in the initial sample, but also had to be commonly associated with codeine misuse on Instagram more broadly. Hashtags and their definitions are listed in Multimedia Appendix 1.

#### Secondary Dataset: Codeine-Related Content Analysis

For analysis, we focused on a total of 100 posts, derived from the first 10 unique posts under each of the 10 hashtags in Multimedia Appendix 1. These posts reflect 73 distinct individuals, of whom 13 posted more than one image in our sample. On average, Instagram users in our sample had 338 posts on their profile (range: 1-3018) and 1270 followers (range: 0-13,000+). Repeat posters were excluded from content analysis as their content was distinct and not relevant to the study question.

We applied grounded theory’s inductive approach for visual content analysis to code our secondary dataset [25]. As with the textual analysis for the primary dataset, this inductive approach to analyzing visual content allowed us to minimize the effect of personal bias surrounding codeine misuse. Through memo writing, we extracted the most salient features of each image or video. For example, a memo for a post demonstrating users preparing codeine for misuse: “user pouring codeine into a Styrofoam cup and mixing it with ice, soda and or hard candy.” Recurrence of certain features or behaviors across multiple posts led us to distill these into themes, such as preparation of codeine. In this way, we produced an emergent coding scheme for our dataset. We discussed a sample of 25 posts (25%, 25/100) to reach consensus on this framework.

After the team developed a final coding scheme, RC and MW completed coding for the remaining posts. After all 100 images or videos were coded, RC then grouped codes under concepts and subsequently categorized these to come up with seven mutually exclusive themes: use-behavior, polysubstance use, preparation of lean, sale, hip-hop, pop culture, and commercialization. Although there is some conceptual overlap between themes (eg, use-behavior and polysubstance use), coders placed posts that were initially double-coded into the theme that most comprehensively captured its content after coming to consensus. Any discrepancies were discussed among team members, and a single theme was agreed upon.

## Results

### Primary Dataset: Data Visualization

**Figure 3:**
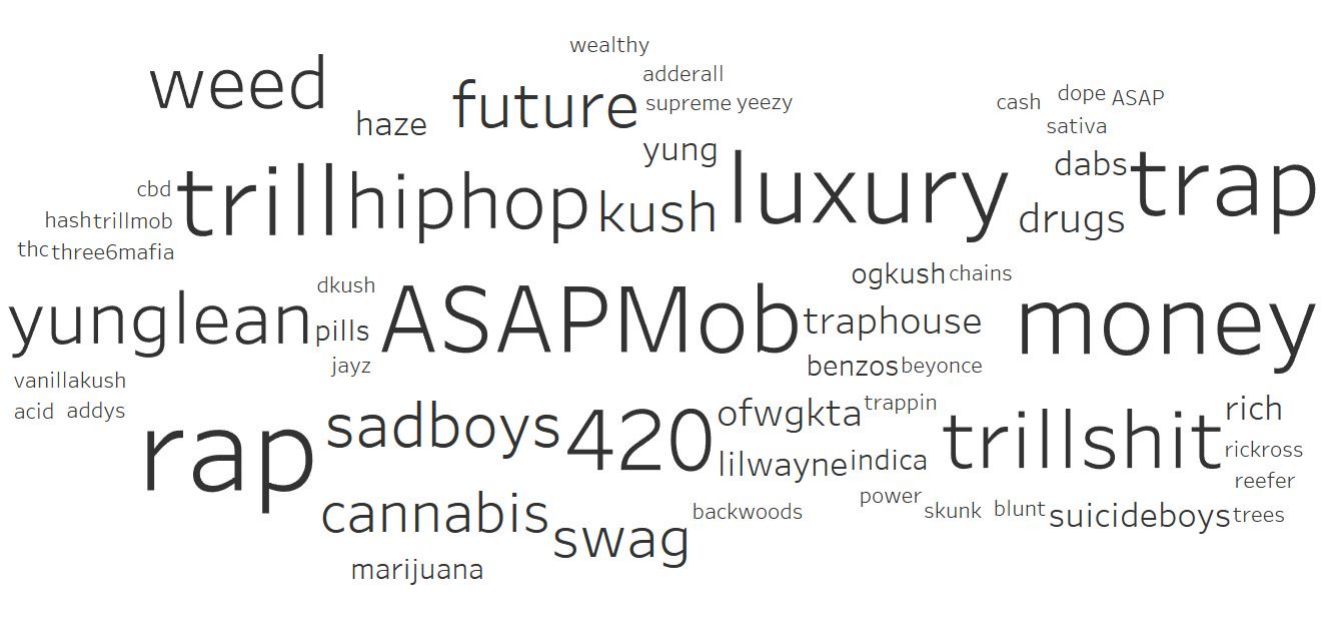
Most common nonopioid-related hashtags associated with #codeine.

Data visualization with prescription opioid hashtags demonstrated terms related to codeinemisuse (Figure 2) and to other activities that co-occurred with these hashtags (Figure 3). Among the terms related to codeine misuse, we came across a number of terms related to processing codeine into the form commonly used in nonmedical consumption, such as lean. Lean, also known as *sizzurp* or *purple drank*, is a concoction consisting of promethazine or codeine cough syrup, ice cubes, and soda, with the optional addition of hard candy [22,26]. Nonopioid hashtags that frequently occurred with codeine-related hashtags revealed a robust relationship between codeine misuse and polysubstance use, especially cannabis use (eg, *weed*, *cannabis*, and *kush*), Additionally, there was a less common association with hip-hop culture (eg, *hip hop* and *rap*; Figure 3).

### Secondary Dataset: Codeine-Related Content Analysis

Definitions and examples of emergent themes are in Multimedia Appendix 2. The greatest proportion of posts were limited to images of codeine or lean in everyday places (*use-behavior*). Posts grouped under *use-behavior* contained images or videos of consuming in their homes, cars, or public places such as the beach. The preparation of codeine from a liquid, cough-syrup formulation into its misused form (ie, lean) was its own theme (*preparation*). Similar to use-behavior, posts grouped under *preparation* depicted the preparation, but not consumption, of lean in an array of common venues. The next most common type of post represented co-occurrence of codeine misuse with recreational alcohol, cannabis, and benzodiazepine consumption (*polysubstance use*). Posts grouped under *polysubstance use* depicted codeine alongside other intoxicants, concurrent consumption, and hashtagged (eg, #cannabis) in the caption. In our thematic framework, *use-behavior* is distinct from *polysubstance use*, in that we only applied the former to posts that showed codeine misuse exclusively, whereas we applied the latter to posts where users represented codeine misuse alongside alcohol or other drugs.

Next were posts those that sought to sell codeine illicitly (*sale*). We grouped posts under *sale* if they included a phone number or a prompt to direct message the user to make an inquiry or purchase. Images or videos with references to popular culture icons (*pop culture)* or those that evoked codeine misuse as a commodity (*commercialization*) were also featured in our sample. The posts grouped under *pop culture* often featured popular culture icons often associated with youth, such as Bart Simpson, Mickey Mouse, and Pokemon. Posts characterized as *commercialization* utilized narratives related to recreational codeine misuse to sell commodities such as a wine cozy with the word *Sizzurp*.

Finally, we also observed posts representing an affinity between codeine misuse in the form of lean and the chopped and screwed genre of hip hop described in previous work [27], as well as a novel association with the trap genre of hip-hop (*hip-hop*). Posts grouped under *hip-hop* featured recognizable recording artists (ie, Future) from this genre in either the imagery or the accompanying text of posts we grouped into this theme. Multimedia Appendix 2 represents definitions, counts, and frequency, as well as examples of each theme. As the sample is 100 posts, counts are equivalent to proportion for each category.

Terms directly referencing codeine misuse in the form of lean were the most prominent across this focused sample. Even after accounting for posts that were drawn into the sample because of a lean-specific hashtag (eg, #siplean, #sizzurp, and #texastea), 47 posts still had references to *lean*. Mentions of the brand names of codeine manufacturers were similarly prominent. Again, after accounting for posts that were draw into the sample because of a brand-specific hashtag (eg, #actavis, #qualitest, and #wockhardt), 28 posts still had references to manufacturers of codeine.

## Discussion

### Principal Findings

Although further large-scale exploration is needed, our preliminary analyses of Instagram posts suggests that codeine misuse may be becoming normalized, commercialized, and ritualized. The most common theme uncovered was a simple visual depiction of lean, which is codeine cough syrup mixed with ice, soda, and occasionally hard candies (ie, Jolly Ranchers). Other prominent themes included depictions of codeine with other substances such as alcohol and cannabis and the preparation of *lean*.

With the majority of posts being limited to displays of preparation or consumption of lean in everyday settings, we can observe that codeine misuse has been integrated into the life of users as normalized, everyday—albeit ritualized—behavior. Moreover, these posts teach and normalize misuse while brazenly transgressing social and legal sanctions. Similarly transgressive, misusers’ appropriation of household pop-culture icons (eg, Bart Simpson or Mickey Mouse or Pokémon) and their integration with lean subculture could make codeine use seem harmless and innocuous, coincident with its hypothesized spread beyond urban ghettos and toward younger, more affluent youth [26,28].

Another common theme in Instagram images was polysubstance use, reflecting findings in the literature that codeine is often combined with marijuana, alcohol, and benzodiazepines, which significantly increases the risk of harm, including overdose mortality, especially among teens [12,13,27,28]. The concurrent misuse of codeine and benzodiazepines is well-documented [29] and is associated with the overdose deaths of notable artists, including DJ Screw himself [22]. There is an urgent need for harm-reduction approaches that emphasize the heightened risks associated these forms of concurrent misuse alongside efforts for primary prevention.

### Comparison With Prior Work

Our findings confirm prior work on opioid misuse using social media data [5-8]. In addition, as previous studies into codeine misuse have described [26,30], some posts in our sample referenced the *chopped and screwed* and *trap* subgenres of hip-hop. Although codeine misuse has existed in Houston since the 1960s and 1970s, its misuse in the form of lean only emerged in the 1980s and 1990s [22]. At this time, local hip-hop artists began incorporating references to its consumption in their music, which came to be known as *chopped and screwed* [22,31]. Chopped and screwed is a technique for remixing songs that was pioneered by DJ Screw in Houston, Texas, during the early 1990s [31]. *Chopped* alludes to the copy-cut-paste sampling technique and stutter effect used to chop up the original song to produce delays and repetition [31]. Although *screwed* was originally a namesake, it is now synonymous with the decreased tempo and thus pitched-down nature that characterizes the style, which the physiological effects of *lean* compliments [31]. Trap music is a subgenre of hip-hop also originating in the Southern United States marked by fast paced beats, synthesizers, and ominous, often nihilistic, lyrical content [32,33]. The term trap originated in Atlanta, Georgia and refers to drug corners and the often inescapable lifestyle (ie, trap) associated with them [33]. Currently, trap music is aggressively marketed and features prominently among Billboard’s top rap songs [34]. Knowing the history and context of codeine misuse is integral for researchers to understand its emergence and spread and ultimately develop appropriate interventions.

Furthermore, our findings demonstrate codeine misuse associated with American popular culture images. This suggests lean is becoming a more mainstream part of popular culture, potentially facilitated by the growing commercialization of trap music over the past decade [34]. Popular culture influences aside, the consumption of codeine in the form of a mixed drink (ie, lean) is inconspicuous and mimetic of a familiar and socially acceptable route of substance (alcohol) ingestion. The affinity between the consumption of codeine in the form of lean and mainstream social alcohol consumption may normalize misuse and promote uptake.

Although lean consumption remains linked to social marginalization [26] since the emergence of lean consumption in the 1980s, the illicit market for codeine has changed dramatically. Once cheaply available over the counter, codeine now requires a prescription or costs upwards of US $1000 a pint on the street [22,35]. Its scarcity has imbued misuse with connotations of wealth, social capital, and exceptionalism. Given the new legal and financial constraints surrounding the procurement of codeine for misuse, it is unlikely that incidence of codeine misuse is occurring among previously studied populations [26]. Rather, we hypothesize that it is now more likely that incidence of misuse is occurring among novel populations who are socially and financially capable of appropriating the identity and lifestyles of mainstream celebrity trap artists (eg, Future) whose images remain prominent in this visual discourse.

The specificity of the narratives (eg, chopped and screwed or trap music) and paraphernalia (eg, double Styrofoam cups) associated with codeine misuse could speak to ritualized or performative activity [36]. Though apparently contradictory, ritualization does not necessarily proscribe normalization. Rather, ritualization is better understood as a process representing initiation into a particular social group that has its own unique sets of norms, practices, and aesthetics [37]. Correctly performing ritualized preparation of codeine cough syrup into the form of lean seems to signify participation or membership in a particular subculture. Similarly, references to trap and chopped and screwed in these representations of misuse speak to these as dominant narratives for codeine misuse. Novel populations of misusers may adopt these images to identify with this particular subculture, perpetuating its use over time [37].

### Limitations

Given the known nonrepresentativeness of social media, our findings should be interpreted as capturing the behavior and narratives of only those misusers who can be identified through the content of their Instagram posts. Furthermore, preliminary analysis was derived from only 2 weeks’ worth of publicly available data from Instagram. Consequently, our sample may not be representative of the full scope of codeine-related posts on Instagram. Relatedly, given the short sample period, there is the possibility that our results are impacted by stationarity. Indeed references to Prince’s death in the spring of 2016 saturated initial attempts at sampling when the study began. When we resumed data collection in summer 2016, no one theme seemed to dominate. However, further larger studies are necessary to confirm any such impact.

However, it was not our intention to undertake a comprehensive study of codeine misuse on Instagram. Rather, we sought to develop a general understanding of Instagram content related to misuse, to fill in current gaps in the literature, and inform future studies of this kind. At a stage where large-scale image analysis is still evolving, we feel that even this initial content analysis is informative for the public health community. Our study is in line with the multiphase approach used in other similar Internet-based research wherein preliminary qualitative analysis on small samples [38,39] are used to inform large-scale automated methodologies [40,41] such as machine learning. However, despite the prevalence of misuse, the paucity of data on this topic requires that any intermediate findings be tested further before deemed useful for the development of much-needed interventions to prevent uptake and curb misuse.

Due to an inability to assess demographics of Instagram users with any sense of reliability or validity, we could not explore whether our findings confirmed previous studies that described the makeup of codeine misusers. To optimize the representativeness of our sample, we extracted posts each day of the week for primary analysis and systematically explored an array of search terms for secondary analysis. Although our sample is not large enough for formal statistical inference, it reveals important trends to explore further and confirms others already known. Future work should make an effort to distinguish the difference, if any, in demographic characteristics of misusers identified through social media content and the general population of misusers derived from survey data.

Although we see an association between a previously defined musical and culture genre and codeine misuse, our findings do not imply any causal relationship between black cultural expression and codeine misuse. Our findings only demonstrate that they co-occur. Though hip-hop may reproduce the phenomena that inform its lyrical content, psychosocial and economic conditions are fundamental causes for both risk behaviors and their aesthetic representation [42].

### Conclusions

The normalization of codeine misuse and emerging associations with popular culture and celebrities suggest that codeine misuse has extended beyond a particular, well-circumscribed subculture. It is paramount to understand how codeine misuse is represented over time to better orient strategies that seek to prevent uptake and curb misuse in both well-known and novel populations.

From this study, it is evident that social media platforms provide forums for crafting and sharing narratives of opioid misuse. As such, platforms such as Instagram provide a lens to gauge perceptions and behaviors surrounding opioid misuse. Given the ubiquity of social media in the lives of adolescents and public nature of this exchange of information, the development of prevention efforts is essential. These data can inform development and testing of countermessaging for these rapidly emerging groups of misusers. We advocate consideration of social media data to inform public health strategies to combat opioid misuse and other shared health behaviors.

## Appendix

Multimedia Appendix 1 Opioid-related hashtags, definitions and examples.

Multimedia Appendix 2 Themes, definitions, and examples.
