# Supplementary material for: Representations of Codeine Misuse on Instagram: Content Analysis
Source: JMIR Public Health Surveill. 2018 Mar 20;4(1):e22. doi: 10.2196/publichealth.8144 (PMC5883072; doi:10.2196/publichealth.8144)
Supplement: Multimedia Appendix 1 [file publichealth_v4i1e22_app1.pdf]

Multi-Media Appendix 1: Opioid-Related Hashtags, definitions and examples

Hashtag and definition	Example	Photo description and caption
#Actavis<br>A pharmaceutical company that discontinued its manufacture of promethazine or codeine cough syrup because of “unlawful and dangerous use of the product…contrary to its approved indication.”	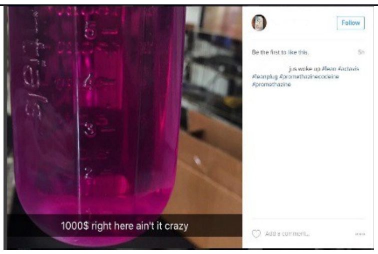	Image of purple liquid in measuring bottle with overlay saying “1000$ right here aint it crazy.”<br>Caption reads: jus woke up #lean #actavis #leanplug #promethazinecodeine #promethazine
#CodeineCrazy<br><br>The title of a popular song by trap artist, Future. It has been viewed more than 18 million times on YouTube.	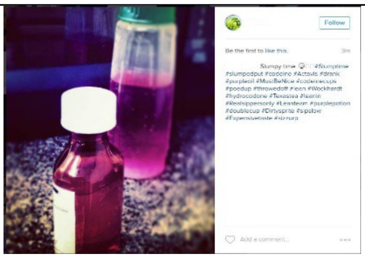	Purple liquid in a bottle and a medicine bottle containing liquid.<br>Caption reads: slumpy time (smiley face emoji) #Slumptime #slumpedout #codeine #Actavis #drank #purpleoil #MustBeNice #codeinecups #poedup #throwedoff #lean #Wockhardt #hydrocodone #Texastea #leanin #Realsippersonly #Leanteam #purplepotion #doublecup #Dirtysprite #sipslow #Expensivetaste #sizzurp
#DoubleCup<br>The signature container for the consumption of lean, made of two nested white Styrofoam cups.	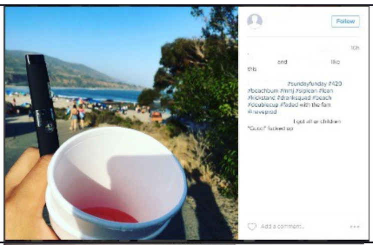	Image of reddish liquid in a double styrofoam cup and a vaporizer pen.<br>Caption reads: #sundayfunday #420 #beachbum #mmj #siplean #lean #kickstand #dranksquad #beach #doublecup #faded with the fam
#OilMobb<br>This term derives from the viscous, oil-like consistency promethazine or codeine cough syrup.	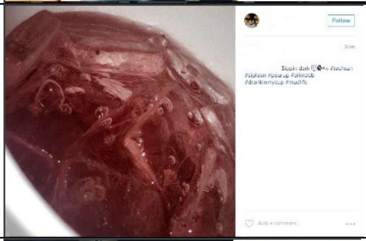	Image of purplish liquid and ice in a styrofoam cup.<br>Caption reads: Sippin dark (cat emoji) (sleep emoji) #techszn #siplean #pourup #oilmobb #drankinmycup #mudlife
#Promethazine<br>The name of a common antihistamine with strong sedative effects. It is often mixed with codeine to treat against cough	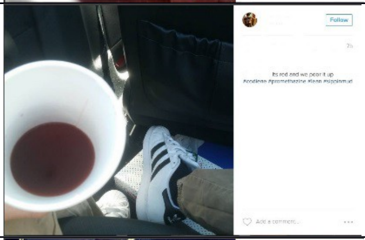	Image of dark liquid I styrofoam cup in car.<br>Caption reads: its red and we poor it up #codeine #promethazine #lean #sippinmud
#Qualitest<br>A pharmaceutical company that manufactures promethazine or codeine cough syrup	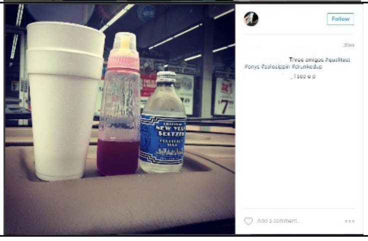	Image of double styrofoam cup, baby bottle with purple liquid, and a bottle of seltzer.<br>Caption reads: three amigos #qualitest #onys #solosippin #drunkedup

#SipLean<br>Refers to the slow consumption of lean and a clothing or accessory brand	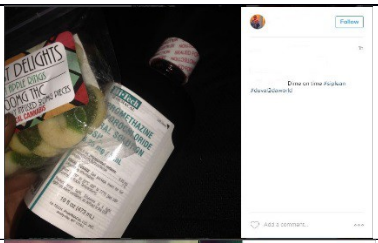	Image of promethazine hydrochloride with apple ring gummy candy.<br>Caption reads: dime on time #siplean #duval2daworld
#Sizzurp<br>Another name for promethazine or codeine cough syrup	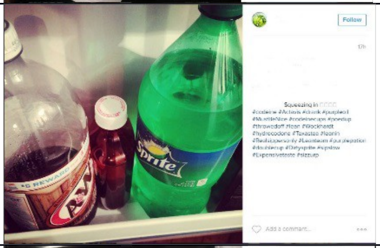	Image of soda and medicine bottle in the shelf of a fridge.<br>Caption reads: squeezing in #codeine #Actavis #drank #purpleoil #MustBeNice #codeinecups #poedup #throwedoff #lean #Wockhardt #hydrocodone #Texastea #leanin #Realsippersonly #Leanteam #purplepotion #doublecup #Dirtysprite #sipslow #Expensivetaste #sizzurp
#TexasTea<br>Another name for promethazine or codeine cough syrup mixed with soda and occasionally hard candy; emphasizes the origins of lean or "purple drank" in Houston, TX	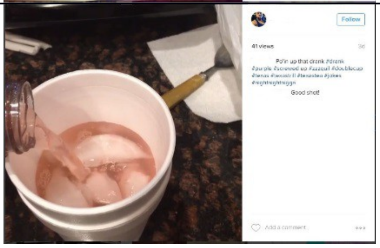	Image of clear liquid being poured into double styrofoam cup with ice and light pink liquid.<br>Caption reads: po'in up that drank #drank #purple #screwed up #zzzquil #doublecup #texas #texastrill #texastea #jokes #nightnightn****
#Wockhardt<br>A pharmaceutical company that manufactures promethazine with codeine cough syrup	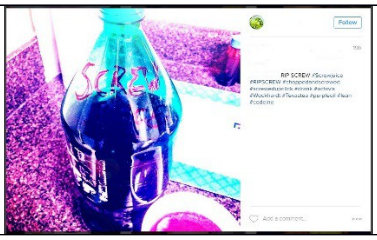	Image of soda bottle with label removed and purple liquid in cup.<br>Caption reads: RIP SCREW #Screwjuice #RIPSCREW #choppedandscrewed #screwedupclick #drank #actavis #Wockhardt #Texastea #purpleoil #lean #codeine
