# Supplementary material for: Representations of Codeine Misuse on Instagram: Content Analysis
Source: JMIR Public Health Surveill. 2018 Mar 20;4(1):e22. doi: 10.2196/publichealth.8144 (PMC5883072; doi:10.2196/publichealth.8144)
Supplement: Multimedia Appendix 2 [file publichealth_v4i1e22_app2.pdf]

Multimedia Appendix 2: Themes, definitions, and examples.

Theme and definition	Frequency n (%)	Example	Photo description and caption
Use-behavior<br>Posts where image solely depicted codeine or lean (a preparation of promethazine or codeine with ice, and soda; occasionally hard candies are added, mostly commonly Jolly Ranchers)	50 (50%)	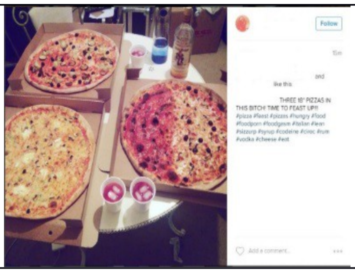	Image of pizzas on a table with three cups of lean, a bottle of Captain Morgan rum, and Ciroc vodka.<br>Caption reads: THREE 18" PIZZAS IN THIS BITCH TIME TO FEAST UP!!!! #pizza #feast #pizzas #hungry #food #foodporn #foodgasm #italian #lean #sizzurp #syrup #syrup #codeine #ciroc #rum #vodka #cheese #eat
Preparation<br>Still images or videos of lean being made.	18 (18%)	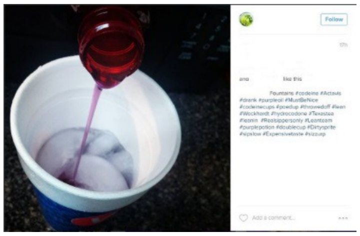	Image of purple syrup being poured into cup with ice.<br>Caption reads: fountains #codeine #Actavis #drank #purpleoil #MustBeNice #codeinecups #poedup #throwedoff #lean #Wockhardt #hydrocodone #Texastea #leanin #Realsippersonly #Leanteam #purplepotion #doublecup #Dirtysprite #sipslow #Expensivetaste #sizzurp
Polysubstance use<br>Posts that displayed the misuse of codeine alongside the use of other substances, such as alcohol and cannabis.	11 (11%)	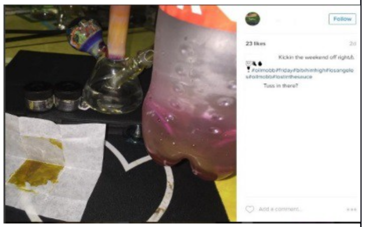	Soda bottle with layer of purple liquid at the bottom, surrounded by a water pipe and cannabis concentrate.<br>Caption reads: kickin the weekend off right (devil emoji) (ok-hand symbol) (fire symbol) #oilmobb #Friday #bitxhimhigh #losangeles #oilmobb #lostinthesauce
Sale<br>Images or videos that displayed bulk quantities of codeine and also had indication in the username, hashtags, or free-text that suggested sale	11 (11%)	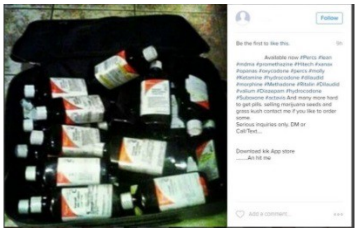	Image of a bag full of promethazine or codeine cough syrup.<br>Caption reads: available now #Percs #lean #mdma #promethazine #Hitech #xanax #opanas #oxycodone #percs #molly #Ketamine #hydrocodone #dilaudid #morphine #Methadone #Ritalin #Dilaudid #morphone #Methadone #Ritalin #dilaudid #valium #Diazepam #hydrocodone #Suboxone #actavis And many more hard to get pills. Selling marijuana seeds and grass kush contact me if you like to order some. Serious inquiries only. DM or Call/text....###-###-#### (number censored)

Pop culture<br>Posts where codeine misuse is associated with pop-culture symbols, icons, or personas	4 (4%)	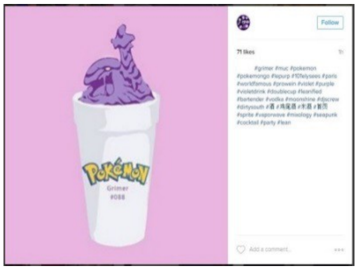	Image of pokemon character, Grimer, coming out of a styrofoam cup.<br>Caption reads: #grimer #muc #pokemon #pokemongo #lepurp #101elysees #paris #worldfamous #prowein #violet #purple #violetdrink #doublecup #leanified #bartender #vodka #moonshine #djscrew #dirtysouth #sprite #vaporwave #mixology #seapunk #cocktail #party #lean
Commercialization<br>Posts that displayed the sale of products bearing common symbols or language associated with codeine misuse. In contrast to sale, commercialization identified posts that sought to sell codeine-related products, not codeine itself.	4 (4%)	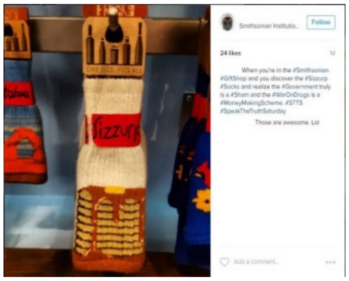	Image of a bottle koozie with the word “Sizzurp” written on it.<br>Caption reads: when you’re in the #Smithsonian #GiftShop and you discover the #Sizzurp #Socks and realize the #Government truly is a #Sham and the #WarOnDrugs is a #MoneyMakingScheme. #STSS #SpeakTheTruthSaturday
Hip-Hop<br>Posts where hip-hop artists were portrayed in the image or video, especially those whose music contain references to codeine misuse (eg, 36 Mafia, Future, and Lil’ Wayne).	3 (3%)	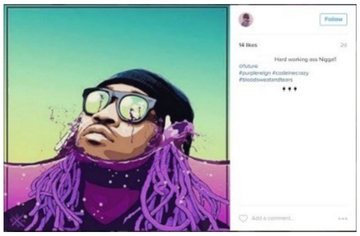	Image of hip-hop artist Future floating in purple liquid.<br>Caption reads: hard working ass N****!!. @future #purplereign #codeinecrazy #bloodsweatandtears
